# Systematic reviews in PRP‐augmented meniscal repair have limited methodological quality: A systematic overview

**DOI:** 10.1002/jeo2.70787

**Published:** 2026-07-03

**Authors:** Ashraf T. Hantouly, Piero Agostinone, Iacopo Romandini, Alexander Sandon, Francesca Zannoni, Sathish Muthu, Khalid Alkhelaifi, Emmanouil Papakostas

**Affiliations:** ^1^ McMaster University Hamilton Ontario Canada; ^2^ Biomedical and Neuromotor Sciences Department University of Bologna Bologna Italy; ^3^ Aspetar Orthopaedic and Sports Medicine Hospital Doha Qatar; ^4^ Clinica Ortopedica E Traumatologica II, IRCCS Istituto Ortopedico Rizzoli Bologna Italy; ^5^ Department of Orthopaedics Orthopaedic Research Group Coimbatore Tamil Nadu India; ^6^ Central Research Laboratory, Meenakshi Medical College Hospital and Research Institute Meenakshi Academy of Higher Education and Research Chennai India

**Keywords:** biological augmentation, meniscal repair, meniscus, platelet‐rich plasma

## Abstract

**Purpose:**

To evaluate the quality of the existing evidence on platelet‐rich plasma (PRP) augmented meniscal repair using a validated appraisal tool, A MeaSurement Tool to Assess systematic Reviews (AMSTAR‐2), and to offer guidance on how the findings of available systematic reviews should be interpreted.

**Study design:**

Systematic review.

**Methods:**

Five databases (PubMed, Web of Science, Embase, OvidMedline and Scopus) were searched from inception until August 2024 for systematic reviews analysing the role of PRP in meniscal repair. Methodological quality was assessed using Oxford Levels of Evidence and AMSTAR 2 grading. This review was conducted with adherence to PRISMA's guidelines for study selection and reporting.

**Results:**

Thirteen systematic reviews, published between 2020 and 2024, met inclusion criteria, analysing four to nine studies each. Nine performed meta‐analyses, with study years ranging from 2014 to 2023. The methodological quality varied, with evidence levels ranging from I to IV. Four reviews conducted sensitivity analysis, four performed subgroup analysis, and one assessed publication bias. All reviews demonstrated critical methodological flaws according to AMSTAR‐2, with 11 (84.6%) rated 'critically low' and two 'low' quality. There was substantial inconsistency in reporting complications and reoperation rates in systematic reviews.

**Conclusion:**

The quality of existing systematic reviews on PRP augmentation for meniscal repair is limited. As a result, there is insufficient evidence to conclude that PRP provides significant clinical or radiological benefit in meniscal repair. Therefore, we recommend cautious interpretation of the available evidence and stress on the need for high quality systematic reviews to pool the available evidence.

**Level of Evidence:**

Level II.

**Abbreviations**
AMSTAR: Assessment of Multiple Systematic Reviews
AMSTAR‐2: Updated Assessment of Multiple Systematic Reviews
Cochrane: Cochrane Collaboration Database
GRADE: Grading of Recommendations Assessment, Development, and Evaluation
IKDC: International Knee Documentation Committee
KOOS: Knee injury and Osteoarthritis Outcome Score
PRISMA: Preferred Reporting Items for Systematic Reviews and Meta‐Analyses
PRP: platelet‐rich plasma
RCT: randomised controlled trial
RoB: risk of bias
VAS: Visual Analog Scale

## INTRODUCTION

Meniscal tears are among the most common injuries encountered in orthopaedics, significantly impairing knee function due to the critical roles of the menisci in load distribution, shock absorption and joint stability [2, 16]. Globally, arthroscopies addressing meniscal pathologies are estimated to reach nearly four million annually [11, 39]. The menisci's limited healing potential poses substantial challenges to successful treatment outcomes [11, 16, 35]. Historically, partial or total meniscectomy was widely adopted as the standard of care [33, 43]. However, the biomechanical consequences of meniscectomy have driven a shift toward repair‐focused strategies [10, 16, 43]. This transition highlights the importance of identifying innovative approaches to improve meniscal healing and long‐term joint health [7, 30]

In recent years, orthobiologics such as platelet‐rich plasma (PRP) have emerged as promising adjuncts to enhance meniscal repair outcomes [22, 33]. PRP is an autologous blood‐derived product obtained via centrifugation, enriched with growth factors such as platelet‐derived growth factor and vascular endothelial growth factor [16, 35]. These factors play pivotal roles in promoting tissue regeneration by enhancing cellular proliferation, angiogenesis, and extracellular matrix production [16, 20, 33, 34]. Preclinical studies have demonstrated PRP's regenerative potential, supporting its application in soft‐tissue repair and regeneration [14, 22, 35]. However, clinical outcomes of PRP‐augmented meniscal repairs remain inconsistent. While some studies report improved healing rates and better patient‐reported outcomes [30, 43], others find no significant benefits, often attributed to heterogeneity in study designs, patient populations, and PRP preparation protocols [7, 11, 16]. Several systematic reviews have attempted to synthesise the available evidence; however, mixed findings, substantial heterogeneity, risk of bias in primary studies, and limitations in reporting quality highlight the need for more robust and standardised research. Therefore, the aim of this study was to evaluate the quality of existing systematic reviews examining PRP's role in enhancing meniscal repairs. The hypothesis was that the quality of these systematic reviews may be insufficient to draw reliable conclusions about the clinical or radiological benefits of PRP augmentation for meniscal repair.

## METHODS

This systematic overview was conducted in accordance with the guidelines of the Back Review Group of Cochrane Collaboration [9] and reported following the Preferred Reporting Items for Systematic Reviews and Meta‐Analyses (PRISMA) [23]. This study was registered in PROSPERO (CRD42025635184).

### Search strategy

An electronic‐based systematic search was conducted covering PubMed, Web of Science, Embase, OVIDMedline and Scopus from inception until August 2024. The focus was on studies evaluating the efficacy of PRP in meniscal repair. Key search terms in the title and abstracts fields was done using 'platelet‐rich plasma', 'PRP', 'platelet‐rich fibrin', 'biologic augmentation', menisc*, 'systematic review' and meta‐analysis while combined with Boolean operators. Manual searches of key references from the included studies were conducted to ensure that all eligible studies were identified.

**Figure 1:**
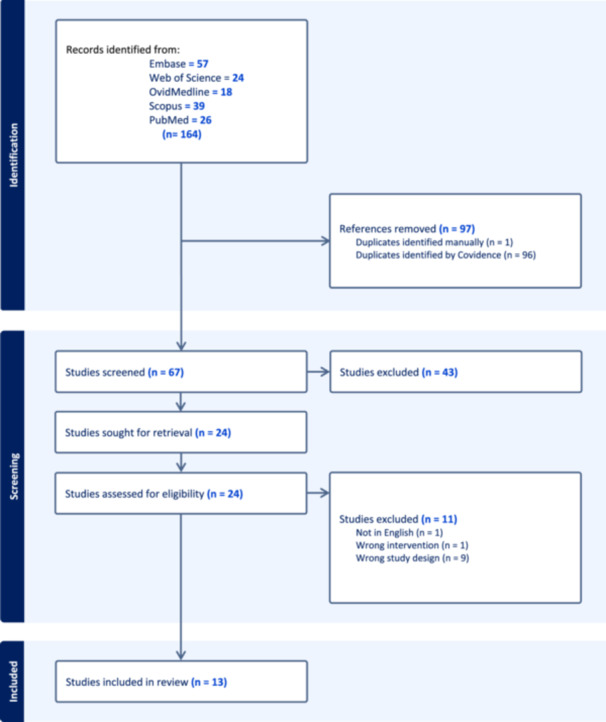
PRISMA flow diagram of inclusion of studies into analysis.

Two reviewers independently conducted a blinded screening of titles and abstracts based on the predefined criteria. Full‐text screening was subsequently performed on eligible studies. Any discrepancies between the reviewers were resolved through the input of a senior author. A PRISMA flow diagram depicting study selection is provided in Figure 1.

### Eligibility criteria

Systematic review study design (with or without meta‐analysis); analysing the role of PRP in meniscal repair; reporting at least one of the outcomes of interest including clinical outcomes, radiological outcomes, complications, or reoperations.

### Exclusion criteria

Narrative reviews, conference abstracts, systematic reviews with less than three studies, case reports, animal model studies, cadaveric studies and non‐English language were excluded.

### Data extraction

Extracted data from the included systematic reviews was done by two reviewers and then cross‐checked to ensure accuracy. Data were was extracted using a priori–designed data collection form (Covidence, Veritas Health Innovation, Melbourne, Australia)These data included first author, publication year, country of origin, date of last literature search, journal name, number and nature of included studies, language restrictions, inclusion/exclusion criteria, databases searched, PROSPERO registration, software used for analysis, subgroup or sensitivity analysis, Grading of Recommendations Assessment, Development, and Evaluation (GRADE) summary, publication bias analysis, conflict of interest, and I^2^ statistic values for each meta‐analysis variable. Disagreements were resolved through consensus.

### Quality assessment

The methodological quality was evaluated using the Oxford Levels of Evidence [29]. Additionally, the updated version of 'A MeaSurement Tool to Assess systematic Reviews' (AMSTAR‐2) was employed to assess methodological robustness [26, 27]. This tool is a 16‐item checklist, three items of which apply only to systematic reviews without meta‐analysis. This tool assigns overall quality ratings of high, moderate, low, or critically low [26, 27]. Following AMSTAR‐2 guidance, particular weight was placed on the checklist's 'critical domains'. Three reviewers independently assessed methodological quality and resolved discrepancies through consensus.

## RESULTS

### Search results

Initial electronic database search yielded 164 articles, reduced to 67 after removing duplicates. Upon title and abstract screening, 43 articles were excluded, leaving 24 for full‐text review. Following full‐text review, 13 systematic reviews were included (Figure 1). The included reviews were published between 2020 and 2024. The number of studies analysed in the included reviews ranged from four to nine, with nine reviews performing meta‐analyses (Table 1). Publication years of included studies ranged from 2014 to 2023 as shown in Table 2. Only five of the included systematic reviews were PROSPERO registered [11, 16, 20, 30, 33].

**Table 1:** Characteristics of the included reviews.

Author	Country	Publication journal	Literature search date	No. of studies included (meta‐analysis studies)	Conclusion	Limitation	Practice implications
Belk et al. [2]	USA	*The Orthopaedic Journal of Sports Medicine*	26 October 2019	6(0)	Meniscal repair with PRP augmentation yields similar clinical outcomes at midterm follow‐up compared with isolated meniscal repair. Further research is needed.	Only six studies were included. Only two studies are of level one evidence. Heterogenous repair techniques. Heterogenous PRP preparations. Heterogenous meniscal tears Heterogenous follow‐up times. Heterogenous definitions of treatment failure and patient distribution.	Similar clinical outcomes at midterm follow‐up compared with conventional meniscus repair
Trams et al. [33]	Poland	*Life 2020*	2005–2020	7 (7)	There is an advantage in the use of PRP compared to control groups. Further research is needed.	Language restriction. Heterogenous PRP preparations. Heterogenous Platelet and leukocyte contents. Level of evidence of included studies I–II. Some studies had high risk of bias.	PRP advantageous in meniscus repair with available limited evidence
Migliorini et al. [22]	Germany	*Journal of Orthopaedics and Traumatology*	August 2021	8 (8)	The evidence does not support the use of PRP as an augment to meniscal repair. At two years follow‐up, meniscal repair with PRP augmentation demonstrated similar functional scores, failure rate and revision rate compared to isolated meniscal repair.	Retrospective design of most of the included studies Heterogenous and limited follow‐up times. Post‐operative rehabilitation was seldom described Inadequate reporting of surgical techniques. Heterogenous PRP preparations.	No additional benefit in augmenting meniscus repair with PRP
Keller et al. [16]	USA	*Knee Surgery, Sports Traumatology, Arthroscopy*	October 2020	7(0)	Patients report significant improvement in functional outcome with PRP augmentation, but the benefit remains questionable. Similarly, the benefit of PRP in regards to revision rates remains questionable.	Selection and publication bias. Heterogenous outcome measures Heterogenous surgical techniques. Heterogenous meniscal tears. Heterogeneity in patients’ age Heterogenous postoperative rehabilitation	Doubtful benefit of PRP in meniscus repair augmentation
Blough et al. [3]	USA	*Journal of Knee Surgery*	20 May 2020	5(0)	There is limited evidence supporting the use of PRP in meniscus repair. However, the current evidence suggests that the highest likelihood for effectiveness of PRP is isolated meniscus repair or in repairs that are not amenable to repair.	Study that analyse different biological augmentation. The major limitation is the methodology of the included studies. The mCMS were mostly poor (mean: 43, range: 17–69), indicating low levels of clinical evidence, poorly con‐ trolled studies, small sample sizes, and nonblinded designs. Another limitation was the wide variety of outcomes meas‐ ures used in different studies. An additional limitation was the heterogeneity of inclusion criteria, repair technique, and rehabilitation protocol, making comparison of like augmentation techniques difficult.	PRP use is best with isolated meniscus repair or tears that are not amenable to repair
Sochacki et al. [30]	USA	*Orthopaedic Journal of Sports Medicine*	27 January 2020	5 (5)	Most studies are of low quality. PRP use in the setting of meniscus repair led to lower failure rates. However, most studies showed no differences in patient reported outcomes.	Heterogenous meniscus tears. Heterogenous patient reported outcomes. Heterogenous surgical techniques. Heterogenous PRP preparations. Heterogenous failure definitions.	PRP augmented meniscus repairs results in lower failure rates
Xie et al. [38]	China	*Medicine (United States)*	1 December 2020	8 (8)	PRP is safe and effective in meniscus repair.	Potential confounding variables such as age, sex, meniscus tears, Heterogenous surgical techniques, Heterogenous or under‐reported PRP preparations All participants included in the study were American or Chinese.	PRP is safe and effective.
Li and Weng [20]	China	*Journal of Orthopaedic Surgery and Research*	2015–2021	9 (9)	PRP use in meniscus repairs led to lower failure rates and improved postoperative pain. However, most studies showed no significant differences in patient‐reported outcomes.	Low number of randomised control trials included. Heterogenous meniscal tears. Heterogenous surgical techniques. Heterogenous PRP preparations. No subgroup or meta regression analyses for the different PRP preparations.	PRP could be recommended in meniscus repair compared with platelet rich fibrin matrix, which was shown to have no benefit in improving functional outcomes.
Haunschild et al. [11]	USA	*Arthroscopy: The Journal of Arthroscopic and Related Surgery*	July 2019	5(0)	The current evidence is insufficient to support the use of PRP in meniscus repair.	Heterogenous follow‐up times. Heterogenous meniscus tears. Heterogenous surgical techniques. Heterogenous PRP preparations.	Insufficient evidence to support PRP in MR
Thahir et al. [32]	UAE, India	*Indian Journal of Orthopaedics*	NR	4 (4)	The current evidence on PRP use in meniscus repair is inconclusive.	Not mentioned clearly in the study	Inconclusive evidence to support PRP in meniscus repair
Wang et al. [35]	China	*Journal of International Medical Research*	NR	6 (6)	PRP has obvious advantages in managing meniscus injury. PRP can reduce postoperative pain, improve knee flexion, and decrease the failure rate. However, in the short‐term follow up, outcome scores did not improve significantly.	No regression analysis No publication bias assessment. Heterogenous PRP preparations.	PRP injection can effectively enhance the efficacy of arthroscopic repair of meniscal injury, reduce the failure rate and severity of pain, and improve active flexion.
Xie et al. [39]	China	*Journal of Orthopaedic Surgery*	19 November 2021	8 (8)	In mid‐term follow up, PRP is worthy of further consideration in improving of meniscus repair, functional score and pain.	Only 9 randomised control trials were included. Heterogenous populations Heterogenous surgical techniques.	PRP is worthy of further consideration in improving the function and pain of patients after MR surgery, and PRP can further improve the healing rate of meniscus repair.
Zaffagnini et al. [43]	Italy	*The Orthopaedic Journal of Sports Medicine*	20 March 2020	5 (5)	PRP is safe and improves survival rate.	Low number of studies were included. Heterogenous patient population Heterogenous treatments. Heterogenous meniscus teras. Heterogenous postoperative rehabilitation protocols. Heterogenous PRP preparations.	PRP safe and useful in improving the survival rate of MR

Abbreviation: PRP, platelet‐rich plasma.

**Table 2:** Primary studies included in each review.

Primary studies	Nature of study	Belk et al. [2]	Trams et al. [33]	Migliorini et al. [22]	Keller et al. [16]	Blough et al. [3]	Sochacki et al. [30]	Xie et al. [38]	Li et al. [20]	Haunschild et al. [11]	Thahir et al. [32]	Wang et al. [35]	Xie et al. [39]	Zaffagnini et al. [43]
Dai [4]	RCS	+	+	+	+	+	+		+	+		+		+
Everhart et al. [7]	PCS	+	+	+	+	+	+		+					+
Griffin et al. [10]	RCS	+	+	+	+	+	+		+	+	+	+		+
Kaminski et al. [14]	RCT	+	+	+	+	+	+	+	+	+		+	+	+
Kaminski et al. [15]	RCT	+	+	+				+	+			+	+	
Pujol [24]	RCS	+	+	+	+	+			+	+	+	+		+
Kemmochi [17]	PCS		+	+	+		+		+	+				
Duif [5]	RCT			+								+	+	
James [13]	Case report				+									
He [12]	RCT							+						
Li [19]	RCT							+					+	
Liu [21]	RCT							+					+	
Zhou [44]	RCT							+						
Shi [28]	RCT							+					+	
Wu [37]	RCT							+					+	
Yang [41]	RCS								+		+			
Bailey [1]	RCS								+					
Yi [42]	RCS										+			
Elnemr [6]	RCT												+	
Qin (2020)*	RCT												+	

Abbreviations: PCS, prospective cohort study; RCS, retrospective cohort study; RCT, randomised control trial.

* Qin 2020 study was included in Xie et al.'s systematic review and meta‐analysis [27] but the referece was not provided.

### Search methodology

While included systematic reviews conducted comprehensive searches, the databases searched varied, with all including PubMed (*n* = 13), followed by Cochrane library (*n* = 11) and Embase (*n* = 10). Other databases searched include Medline (*n* = 3), Scopus (*n* = 3), Clinictrials.gov (*n* = 3), Web of Science (*n* = 2), China National Knowledge Infrastructure database (*n* = 2), Google Scholar (*n* = 1) and Cumulated Index in Nursing and Allied Health Literature (*n* = 1). Further details on the search methodology employed by the included studies were presented in Table 3.

**Table 3:** Search methodology used by each study.

Search parameters	Belk et al. [2]	Trams et al. [33]	Migliorini et al. [22]	Keller et al. [16]	Blough et al. [3]	Sochacki et al. [30]	Xie et al. [38]	Li et al. [20]	Haunschild et al. [11]	Thahir et al. [32]	Wang et al. [35]	Xie et al. [39]	Zaffagnini et al. [43]
Publication language restriction	Only English	Only English	English, German, Italian, French and Spanish	Only English	Only English	Only English	None	Only English	Only English	Only English	Not Mentioned	None	Only English
PubMed	X	X	X	X	X	X	X	X	X	X	X	X	X
Medline					X			X	X				
Embase	X	X	X	X			X	X	X	X	X	X	
Cochrane library	X	X		X		X	X	X	X	X	X	X	X
Web of Science			X					X					
Scopus						X				X			X
Google Scholar			X										
CNKI database							X					X	
CINAHL				X									
Clinicaltrials.gov		X					X					X	

Abbreviations: CINAHL, Cumulated Index in Nursing and Allied Health Literature; CNKI, China National Knowledge Infrastructure.

### Methodological quality and reported outcomes

Using Oxford Levels of Evidence, included studies were classified as Level I–IV due to the variability in primary study designs (Table 4). One study assessed for publication bias in their analysis. Four studies performed sensitivity analysis and four studies conducted subgroup analyses.

**Table 4:** Methodological information of each study.

Methodology	Belk et al. [2]	Trams et al. [33]	Migliorini et al. [22]	Keller et al. [16]	Blough et al. [3]	Sochacki et al. [30]	Xie et al. [38]	Li et al. [20]	Haunschild et al. [11]	Thahir et al. [32]	Wang et al. [35]	Xie et al. [39]	Zaffagnini et al. [43]
Primary study design	Mixed	Mixed	Mixed	Mixed	Mixed	Mixed	RCT	Mixed	Mixed	Mixed	Mixed	RCT	Mixed
Level of evidence	III	III	III	IV	IV	III	I	III	III	IV	III	I	III
Software used	NR	RevMan 5.3	RevMan 5.3	NR	NR	RevMan 5.3	NR	RevMan 5.4 & STATA	Excel	RevMan 5.3	RevMan 5.3	NR	Excel
GRADE used	No	No	No	No	No	No	No	No	No	Yes	No	No	No
Sensitivity analysis	No	No	No	No	No	No	Yes	Yes	No	No	Yes	Yes	No
Subgroup analysis	No	Yes	No	No	No	No	Yes	Yes	No	No	No	Yes	No
Publication bias	No	No	Yes	No	No	No	No	No	No	No	No	No	No

Abbreviations: GRADE, Grading of Recommendations Assessment, Development and Evaluation system; NR, not reported; RCS, retrospective cohort study.

Based on AMSTAR‐2 assessment, all included studies demonstrated critical methodological limitations. Of the 13 included studies evaluated, 11 (84.6%) were rated as 'critically low' quality, while the remaining two were classified as 'low' quality. Compliance with key methodological domains was variable. The most consistently adhered to domains were the use of a clearly defined PICO framework (13/13), duplicate study selection (12/13), and duplicate data extraction (11/13) (Questions 1, 5 and 6 in Table 5). Conversely, providing a list of the excluded studies and reporting of funding sources for the primary studies (Questions 7 & 10 in Table 5) were absent across all studies, representing the least adhered‐to domain. A detailed breakdown of AMSTAR‐2 ratings is provided in Table 5.

**Table 5:** AMSTAR 2 tool for included studies.

Items	Belk et al. [2]	Blough et al. [3]	Haunschild et al. [11]	Keller et al. [16]	Li et al. [20]	Migliorini et al. [22]	Sochacki et al. [30]	Thahir et al. [32]	Trams et al. [33]	Wang et al. [35]	Xie et al. [38]	Xie et al. [39]	Zaffagnini et al. [43]
1. Did the research questions and inclusion criteria for the review include the components of PICO?	Yes	Yes	Yes	Yes	Yes	Yes	Yes	Yes	Yes	Yes	Yes	Yes	Yes
2. Did the report of the review contain an explicit statement that the review methods were established prior to the conduct of the review and did the report justify any significant deviations from the protocol? **	No	No	Yes	Yes	Yes	No	Yes	No	Yes	No	No	No	No
3. Did the review authors explain their selection of the study designs for inclusion in the review?	No	Yes	Yes	Yes	Yes	No	Yes	Partial yes	Partial yes	Partial yes	Partial yes	Yes	Yes
4. Did the review authors use a comprehensive literature search strategy? **	Partial yes	Partial yes	Partial yes	Partial yes	Yes	Partial yes	Partial yes	Partial yes	Yes	Partial yes	Partial yes	Partial yes	Yes
5. Did the review authors perform study selection in duplicate?	Yes	Yes	Yes	Yes	Yes	Yes	No	Yes	Yes	Yes	Yes	Yes	Yes
6. Did the review authors perform data extraction in duplicate?	No	Yes	Yes	Yes	Yes	Yes	No	Yes	Yes	Yes	Yes	Yes	Yes
7. Did the review authors provide a list of excluded studies and justify the exclusions? **	No	No	No	No	No	No	No	No	No	No	No	No	No
8. Did the review authors describe the included studies in adequate detail?	Partial yes	Partial yes	Partial yes	Partial yes	yes	Yes	Yes	Partial yes	Yes	Partial yes	Yes	Yes	Partial yes
9. Did the review authors use a satisfactory technique for assessing the risk of bias (RoB) in individual studies that were included in the review? **	Yes	Yes	Yes	Partial yes	yes	Yes	Yes	Yes	Partial yes	Yes	Yes	Yes	Yes
10. Did the review authors report on the sources of funding for the studies included in the review?	No	No	No	Yes	No	No	No	No	No	No	No	No	No
11. If meta‐analysis was performed, did the review authors use appropriate methods for statistical combination of results? **	No MA	No MA	No MA	No MA	Yes	Yes	Yes	Partial yes	Yes	Yes	Yes	Yes	Yes
12. If meta‐analysis was performed, did the review authors assess the potential impact of RoB in individual studies on the results of the meta‐analysis or other evidence synthesis?	No MA	No MA	No MA	No MA	Partially yes	No	No	No	Partial yes	No	Partial yes	No	Partial yes
13. Did the review authors account for RoB in primary studies when interpreting/discussing the results of the review? **	Yes	Yes	Yes	No	Yes	No	No	No	Yes	Yes	Yes	Yes	Yes
14. Did the review authors provide a satisfactory explanation for, and discussion of, any heterogeneity observed in the results of the review?	No	No	Yes	Partially yes	Yes	Yes	Yes	Yes	Partial yes	Partial yes	Yes	Yes	Yes
15. If they performed quantitative synthesis did the review authors carry out an adequate investigation of publication bias (small study bias) and discuss its likely impact on the results of the review? **	No MA	No MA	No MA	No MA	No	Yes	No	Partial yes	Partial yes	No	No	No	No
16. Did the review authors report any potential sources of conflict of interest, including any funding they received for conducting the review?	Yes	Yes	Yes	Yes	Yes	Yes	Yes	No	Yes	Yes	Yes	Yes	Yes
Overall confidence rating	Critically low	Critically low	Low	Critically low	Critically low	Critically low	Critically low	Critically low	Critically low	Critically yow	Critically low	Critically low	Low

Abbreviation: AMSTAR‐2, A MeaSurement Tool to Assess systematic Reviews.

*Note*: ‘Critical domains’ noted with a double asterisk (**).

Radiological outcomes were reported in only two of the included reviews (15.4%), whereas functional outcomes were documented in seven of the 13 studies (53.8%). Revision rates were addressed in two reviews (15.4%), and complication rates were reported in five studies (38.5%) (Table 6).

**Table 6:** Reporting of outcomes of interest in the included reviews.

Outcome variables	Belk et al. [2]	Trams et al. [33]	Migliorini et al. [22]	Keller et al. [16]	Blough et al. [3]	Sochacki et al. [30]	Xie et al. [38]	Li et al. [20]	Haunschild et al. [11]	Thahir et al. [32]	Wang et al. [35]	Xie et al. [39]	Zaffagnini et al. [43]
Radiological improvement	NA	NA	NA	NA	NA	NA	Healing rate 66% Subgroup America 0% China 34% Differences 71,3%	NA	NA	NA	NA	Healing rate 66% Subgroup America 0% China 34% Differences 71,3%	NA
Functional improvement	NA	96% subgroup 31,7%	IKDC 100% VAS 84% Lysholm 79%	NA	NA	NA	VAS 9% Lysholm 84% After sensivity 15%	IKDC 93% subgroup 95% Lysholm 69% Subgroup 0% VAS 57% KOOS pain 99% KOOS Symptom 95% KOOS ASL 99% KOOS Sport/recreation 99% KOOS‐QoL 98%	NA	VAS 100% Lysholm 79% IKDC 0% ROM 42%	IKDC 97% Lysholm 87% VAS 98% Active flexion 0%	VAS 9% Lysholm 84% After sensivity 15%	NA
Revision rate	NA	0% subgroup 0%	58%	NA	NA	NA	NA	NA	NA	NA	NA	NA	NA
Complication rate	NA	NA	Failure 0%	NA	NA	Failure 32%	NA	Failure 0%	NA	Failure 0%	Failure 31%	NA	NA

Abbreviations: IKDC, International Knee Documentation Committee; KOOS, Knee injury and Osteoarthritis Outcome Score; NA, not applicable; QoL, quality of life; ROM, range of motion; VAS, Visual Analog Scale.

## DISCUSSION

The main finding in this study was that the overall quality of systematic reviews assessing PRP augmentation for meniscal repair is low to critically low. None of the included reviews achieved moderate or high methodological quality as per AMSTAR‐2, reflecting significant flaws in study design, execution, and reporting. This underscores the challenges of drawing reliable conclusions from reviews that synthesise data from low‐quality, heterogeneous primary studies. The lack of robust evidence limits the ability to make definitive clinical recommendations regarding the efficacy of PRP in meniscal repair.

Radiological improvement, a key outcome and surrogate for biological healing, was either sparsely or inconsistently reported across the included reviews. Faster or superior healing is crucial for PRP to be considered effective, but the available evidence does not consistently support such claims. For instance, studies have shown variability in radiological outcomes [36], with limited subgroup analyses to explain these differences. Moreover, factors such as heterogeneity in PRP preparation methods and patient‐specific variables may confound interpretations of radiological findings. Previous research highlights the need for standardised imaging protocols and clear definitions of healing to improve the reliability of these assessments [8, 25, 40].

Pain and functional improvement were assessed using tools such as IKDC, VAS, KOOS, Lysholm and range of motion. While some reviews reported modest benefits, others demonstrated no significant differences between PRP‐augmented and standard repairs. These discrepancies can be attributed to heterogeneity in outcome measures, follow‐up durations, and patient populations. Furthermore, questions remain regarding the sensitivity of these tools to detect clinically meaningful differences in functional recovery. Tapasvi et al found no correlation between healing status of the meniscus and any KOOS subscale, IKDC or Lysholm score [31]. The lack of consistent functional improvement calls into question PRP's utility in routine clinical practice.

The revision rate, a critical marker of long‐term repair success, was reported in only one systematic review, significantly limiting the capacity to evaluate PRP's impact in reducing reoperation risk. Revision surgery is often influenced by multiple factors, including surgical technique, patient compliance, and baseline tear characteristics [7, 18, 34]. The paucity of data on this outcome highlights the need for future research to address this gap comprehensively.

Complication rates were similarly underreported, with inconsistent definitions and outcomes across studies. While some reviews suggested lower failure rates with PRP, these findings were not universally supported and were confounded by differences in PRP application techniques and patient selection criteria. Safety profiles remain a critical consideration for clinical adoption, and the lack of standardised reporting in this domain underscores the need for rigorous prospective studies.

This study has several limitations that must be acknowledged. The heterogeneity in the methods, outcomes, and patient populations of the included systematic reviews complicates interpretation and limits generalisability. The absence of standardised reporting across the included systematic reviews posed significant challenges. Outcome measures such as radiological improvement, functional scores, and complication rates were inconsistently defined and reported, making it difficult to draw meaningful comparisons. Additionally, key outcomes such as revision rates were either underreported or entirely missing, further limiting the scope of our analysis. Our study did not directly assess the primary studies included within the systematic reviews but instead relied on the synthesised results presented in those reviews. As a result, any inaccuracies, biases, or methodological flaws in the included primary studies could have been carried over into our analysis without being addressed.

Despite these limitations, this study highlights critical gaps in the literature, emphasising the urgent need for higher‐quality primary studies and systematic reviews with rigorous methodologies to establish the clinical efficacy and safety of PRP in meniscal repair. The critically low quality of existing systematic reviews combined with inconsistencies in radiological and functional outcomes, as well as revision and complication rates, underscores the necessity for robust evidence to support clinical practice. Future efforts should focus on addressing the methodological shortcomings identified here to provide a stronger foundation for evidence‐based decision‐making.

### Conclusion

The quality of existing systematic reviews on PRP augmentation for meniscal repair is limited. As a result, there is insufficient evidence to conclude that PRP provides significant clinical or radiological benefit in meniscal repair. Therefore, we recommend cautious interpretation of the available evidence and stress on the need for high quality systematic reviews to pool the available evidence.

## AUTHOR CONTRIBUTIONS

All authors had substantial contribution to the design of the study. Francesca Zannoni, Piero Agostinone, Iacopo Romandini, Sathish Muthu and Ashraf T. Hantouly collected data. Ashraf T. Hantouly, Piero Agostinone and Iacopo Romandini and Alexander Sandon participated in data interpretation. Ashraf T. Hantouly, Francesca Zannoni, Iacopo Romandini, Piero Agostinone and Alexander Sandon participated in manuscript drafting. Ashraf T. Hantouly, Iacopo Romandini and Francesca Zannoni prepared the final version of the manuscript which reviewed by all authors. Khalid Alkhelaifi and Emmanouil Papakostas supervised the whole project, helped prepare the manuscript and reviewed the final manuscript.

## FUNDING INFORMATION

The authors have no funding to report.

## CONFLICT OF INTEREST STATEMENT

The authors declare no conflicts of interest.

## ETHICS STATEMENT

The authors have nothing to report.

## Supporting information

Supp Table 1. List of the excluded studies.

## Data Availability

The authors have nothing to report.
