# Supplementary material for: Systematic reviews in PRP‐augmented meniscal repair have limited methodological quality: A systematic overview
Source: J Exp Orthop. 2026 Jul 3;13(3):e70787. doi: 10.1002/jeo2.70787 (PMC13331554; doi:10.1002/jeo2.70787)
Supplement: Supplementary file 1 — Supp Table 1. List of the excluded studies. [file JEO2-13-e70787-s001.docx]

**Supplementary Table 1. List of the excluded studies.**

| Study | Reason for Exclusion |
| --- | --- |
| Cetik, R.M., Familiari, F. & Huri, G. Can Meniscal Healing Improve by Interventions in Adults: Systematic Review of Randomized-Controlled Trials. *SN Compr. Clin. Med.* **4**, 206 (2022). https://doi.org/10.1007/s42399-022-01290-2 | <3 eligible PRP studies |
| Conte P., et al. Orthobiologic injections for treating degenerative meniscus lesions: a matter of facts? Ten years of clinical experience in a systematic review. J Cartilage and Joint Preservation. Jun 2023. Vol 3. Https://doi.org/10.1016/j.jcjp.2023.100104 | Conservative management |
| Elphingstone JW, Alston ET, Colorado BS. Platelet-rich plasma for nonoperative management of degenerative meniscal tears: A systematic review. J Orthop. 2024 Mar 12;54:67-75. doi: 10.1016/j.jor.2024.03.009. | Conservative management |
| Filardo G, Kon E, Roffi A, Di Matteo B, Merli ML, Marcacci M. Platelet-rich plasma: why intra-articular? A systematic review of preclinical studies and clinical evidence on PRP for joint degeneration. Knee Surg Sports Traumatol Arthrosc. 2015 Sep;23(9):2459-74. doi: 10.1007/s00167-013-2743-1. | <3 eligible PRP studies |
| Gopinatth V, Batra AK, Chahla J, Smith MV, Matava MJ, Brophy RH, Knapik DM. Degenerative Meniscus Tears Treated Nonoperatively With Platelet-Rich Plasma Yield Variable Clinical and Imaging Outcomes: A Systematic Review. Arthrosc Sports Med Rehabil. 2024 Feb 23;6(2):100916. doi: 10.1016/j.asmr.2024.100916. | Conservative management |
| Mazy D, Wang J, Dodin P, Lu D, Moldovan F, Nault ML. Emerging biologic augmentation strategies for meniscal repair: a systematic review. BMC Musculoskelet Disord. 2024 Jul 13;25(1):541. doi: 10.1186/s12891-024-07644-2. | <3 eligible PRP studies |
| Moran CJ, Busilacchi A, Lee CA, Athanasiou KA, Verdonk PC. Biological augmentation and tissue engineering approaches in meniscus surgery. Arthroscopy. 2015 May;31(5):944-55. doi: 10.1016/j.arthro.2014.11.044. | <3 eligible PRP studies |
| Redler LH, Thompson SA, Hsu SH, Ahmad CS, Levine WN. Platelet-rich plasma therapy: a systematic literature review and evidence for clinical use. Phys Sportsmed. 2011 Feb;39(1):42-51. doi: 10.3810/psm.2011.02.1861. | Different pathologies included |
| Sakti M, Paturusi IA, Singjie LC, Kusuma SA. The Use of Platelet-Rich Plasma Augmentation in Meniscus Repair Results in a Lower Failure Rate than in the Control Group: A Systematic Review From Meta-analysis. Arthrosc Sports Med Rehabil. 2024 Apr 9;6(4):100934. doi: 10.1016/j.asmr.2024.100934. | Wrong study design |
| Twomey-Kozak, John et al. Meniscus Repair and Regeneration. 2020. Clinics in Sports Medicine, Volume 39, Issue 1, 125 – 163. DOI: 10.1016/j.csm.2019.08.003 | Wrong study design |
| Wei, D. et al. Arthroscopic Surgery Combined with Platelet-Rich Plasma for Meniscus Injury: A Meta analysis.  China journal of orthopaedics and traumatology 2021;34(9):879-886  2021. DOI: 10.12200/j.issn.1003-0034.2021.09.018 | Non-English |
